# Cellular Logistics and Synaptic Vesicle Vulnerability in Major Depressive Disorder and Amyotrophic Lateral Sclerosis Comorbidity: Insights From Nicotinamide Mononucleotide Rescue and Transcriptome-Wide Association Study Integration

**DOI:** 10.7759/cureus.113549

**Published:** 2026-07-28

**Authors:** Ngo Cheung

**Affiliations:** 1 Psychiatry, Cheung Ngo Medical Limited, Hong Kong, HKG

**Keywords:** amyotrophic lateral sclerosis, autophagy, endocytosis, major depressive disorder, microglia, nad+, nmn, s-predixcan, synaptic pruning, synaptic vesicle cycle

## Abstract

Background: Major depressive disorder (MDD) and amyotrophic lateral sclerosis (ALS) are usually treated as unrelated, yet depressive symptoms occur in a substantial minority of people with ALS and may appear early. These symptoms are heterogeneous and may reflect syndromal MDD, psychological and functional burden, fatigue, apathy, pseudobulbar affect, frontotemporal involvement, sleep or respiratory disturbance, medication effects, or shared affective vulnerability. A proposed pruning-continuum model suggests both disorders may share vulnerability in microglia-mediated synaptic pruning, with ALS amplified by autophagy and protein-quality-control failure and MDD by RNA-processing, stress, and immune dysregulation. We performed an exploratory secondary transcriptome-wide association study (TWAS)/pathway-integration analysis to test whether predefined nicotinamide mononucleotide (NMN)-nominated pathways map onto this vulnerability.

Methods: We integrated precomputed S-PrediXcan outputs for MDD and ALS across available brain-relevant tissues. Ten Kyoto Encyclopedia of Genes and Genomes (KEGG) pathways were predefined from a prior re-analysis of NMN-associated transcriptional programs in aged mouse metabolic tissues. Mouse-derived candidates were represented by human ortholog symbols before the human TWAS screen. The analysis tested nominated pathways rather than the 35-gene NMN-robust list as a standalone set. Cross-tissue screening used Stouffer Z aggregation, tissue-level Wilcoxon testing, competitive permutation testing, percentile bootstrap intervals, pairwise disease statistics, Levene variance tests, concordance measures, and leave-one-out sensitivity analysis. No analysis was treated as confirmatory or evidence of causal mediation.

Results: MDD showed the strongest Stouffer-based exploratory signal in the synaptic vesicle cycle pathway, with a meta-across-tissue Stouffer Z of 3.41 and a wide bootstrap 95% confidence interval of −0.46 to 7.40. This signal did not survive competitive permutation testing (p = 0.1222) or Wilcoxon testing (p = 0.1926). The strongest tissue-level result occurred in the amygdala (Z = 4.057; nominal Wilcoxon p = 0.0093), although tissue-level permutation testing was not performed in the multi-gene-set run. ALS showed no significant meta-across-tissue enrichment among the 10 nominated pathways but displayed candidate gene-level signals in autophagy, endosomal, and vesicle-related genes, including TBK1 and C9orf72. Exploratory Levene tests indicated variance heterogeneity in the regulation of the actin cytoskeleton, endocytosis, and neuroactive ligand-receptor interaction; the actin cytoskeleton and endocytosis remained significant in pooled global false discovery rate (FDR) analysis. Fourteen genes were influential in at least two focus pathways, including EGF, KNG1, FGF8, RAC1, PAK1, PAK2, RAF1, MAPK1, and FGFR1.

Conclusions: These findings are hypothesis-generating. MDD and ALS may stress overlapping cellular logistics processes while engaging largely different genes. MDD showed the strongest exploratory pathway-level signal in synaptic vesicle biology, whereas ALS showed candidate gene-level coherence in autophagy and endosomal processes without significant meta-pathway enrichment. NMN/NAD+ repletion is not established as a treatment for MDD, ALS, or their comorbidity. These findings generate hypotheses about NAD+-linked cellular stress pathways for future preclinical and clinical studies.

## Introduction

Clinical problem

Major depressive disorder (MDD) and amyotrophic lateral sclerosis (ALS) sit in different parts of medicine. One is usually encountered in psychiatry; the other in neurology. One is defined by low mood, anhedonia, cognitive slowing, and altered stress responsivity; the other, by progressive loss of upper and lower motor neurons, weakness, paralysis, and eventual respiratory failure. Yet the clinical boundary between them is not as clean as the textbooks suggest. Depression is common in ALS, with systematic reviews and clinical studies placing its prevalence roughly between 10% and 34% [1-3]. In some patients, low mood, loss of drive, emotional flattening, or cognitive fatigue appears early enough that it cannot be comfortably dismissed as a reaction to the diagnosis.

That timing is clinically important. If depression in ALS were always a psychological response to devastating news, then treatment would remain largely supportive and symptomatic. But if a subset of mood symptoms reflects early involvement of shared synaptic, immune, or metabolic biology, then depression could become a clue to disease biology rather than only a comorbidity to be managed. This distinction matters because disease-modifying options remain limited in ALS, while many people with MDD, especially treatment-resistant depression, continue to receive treatments that were not designed around circuit repair, microglial biology, or cellular metabolism. A mechanistic bridge between ALS mood symptoms and MDD could therefore open a practical translational space: earlier screening, better stratification, and adjunctive interventions aimed at upstream cellular vulnerability rather than late downstream symptoms.

Depressive symptoms in ALS should not, however, be treated as a unitary phenotype. Clinically, it is important to distinguish MDD meeting syndromal criteria; depressive symptoms secondary to diagnosis-related distress, disability, loss of autonomy, fatigue, sleep disruption, respiratory insufficiency, medication effects, or social isolation; apathy or reduced initiation; pseudobulbar affect; frontotemporal cognitive or behavioral involvement; and a possible biologically shared affective vulnerability in a subset of patients. The present analysis cannot distinguish these presentations. Early screening and longitudinal monitoring are warranted, but early mood symptoms should not be interpreted as proof of prodromal shared MDD-ALS biology.

Existing framework

In a prior analysis by the present author, a proposed microglial pruning continuum was used to link MDD and ALS [4]. In that model, synaptic pruning vulnerability acts as a shared substrate. ALS then diverges toward motor neuron degeneration through autophagy collapse, protein aggregation, and endosomal stress, while MDD diverges toward stress-sensitive synaptic plasticity failure through RNA processing, immune activation, and chronic inflammatory signaling. This remains a proposed framework rather than an established disease model. It helps reconcile an apparent contradiction. At the genome-wide level, MDD and ALS do not show strong shared polygenic architecture; the genetic correlation reported in that prior analysis was near zero. Yet at the pathway level, synaptic pruning, complement biology, microglial recognition, and synaptic plasticity repeatedly appear as plausible points of contact.

This distinction between genome-wide overlap and pathway-level convergence is not trivial. Complex disorders can share biological pressure points without sharing the same common variants across the genome. In ALS, large-scale genetic work has emphasized neuron-specific biology, vesicle-mediated transport, autophagy, and protein homeostasis [5]. In MDD, a large trans-ancestry Genome-Wide Association Study (GWAS) has implicated many loci with enrichment in brain cell types and pharmacologically relevant pathways, reflecting broad polygenicity rather than single-gene causation [6]. A pathway-level framework, therefore, fits the biology better than a simple expectation of high genome-wide genetic correlation.

Gap and opportunity

The pruning-continuum model is useful, but it remains broad. “Synaptic pruning” can mean complement tagging, microglial engulfment, cytoskeletal motility, vesicle recycling, receptor internalization, lysosomal degradation, or synaptic-vesicle release machinery. Each of these steps is biologically different, and some are more druggable than others. A more granular model is needed if the framework is to move from interpretation toward intervention.

This is where cellular logistics becomes a useful organizing idea. Neurons and microglia depend on the tightly regulated movement of receptors, vesicles, proteins, lipids, mitochondria, and damaged cellular material. Endocytosis, recycling endosomes, synaptic vesicle turnover, actin remodeling, autophagy, and metabolic substrate switching are not isolated pathways. They form the infrastructure that keeps synapses stable under stress. If this infrastructure is genetically fragile in MDD and ALS, then the comorbidity between mood disturbance and motor neuron vulnerability may reflect stress on a shared logistics layer rather than direct identity between the two diseases.

Nicotinamide mononucleotide biology as a bridge

A separate author-derived re-analysis of GSE85718 [7], based on the long-term nicotinamide mononucleotide (NMN) aging study by Mills and colleagues [8], identified 35 genes reported as robustly rescued by NMN in at least two of three peripheral metabolic mouse tissues. Because these genes originated from aged mouse metabolic tissues rather than human brain tissue, the current transcriptome-wide association study (TWAS) analysis was used only as a human central nervous system readout of potentially related biological processes, not as evidence that NMN directly rescues the identified human brain pathways.

The most biologically notable genes included RAB11A, CPT2, MAP2K2, MYO15, GALR1, SLC13A5, TRP53INP2, APBA2, ACD, ZFAND2B, and others. No single gene was rescued across all three tissues, suggesting that NMN response was tissue-dependent rather than universal. Still, the pathway pattern was coherent. RAB11A is a central regulator of recycling-endosome organization and provides a direct link to the cellular-logistics concept: recycling-endosome machinery controls the return of receptors and membrane proteins to the cell surface and is relevant to synaptic receptor recycling and membrane handling in immune cells [9,10]. CPT2 pointed toward mitochondrial fatty-acid oxidation and PPAR-linked substrate handling. MAP2K2 pointed toward MAPK/RAS signaling. Other genes suggested links to vesicular trafficking, the actin cytoskeleton, autophagy-related regulation, neuroactive ligand-receptor biology, and synaptic vesicle-associated processes.

This pattern is relevant because NAD+ biology is closely tied to aging, mitochondrial metabolism, cellular stress responses, and tissue resilience [11]. NMN is not established as a treatment for MDD, ALS, or their comorbidity. The prior NMN analysis is better regarded as a biological probe that nominated processes for further investigation than as evidence of therapeutic rescue in human brain disease. If the NMN-associated transcriptional programs overlap with genetically fragile pathways in brain disorders, they may help identify modifiable biology. In other words, NMN biology may act less as a direct disease-specific therapy and more as a probe of pathways that become vulnerable with aging, stress, synaptic remodeling, and impaired cellular repair.

Study aim and translational framing

The primary objective of the present exploratory secondary analysis was to test whether 10 predefined NMN-nominated Kyoto Encyclopedia of Genes and Genomes (KEGG) pathways show pathway-level signals in brain-relevant human TWAS outputs for MDD and ALS. The secondary objectives were to describe tissue-level patterns, cross-disease divergence, variance heterogeneity, concordance, and gene-level sensitivity to leave-one-out removal. The human brain TWAS results were used as a CNS-oriented test of pathways first nominated from a peripheral/metabolic mouse NMN experiment. The analysis was not designed to establish causal mediation, direct NMN rescue, a shared therapeutic target, or a clinical biomarker.

## Materials and methods

Study design and data sources

**Figure 1:**
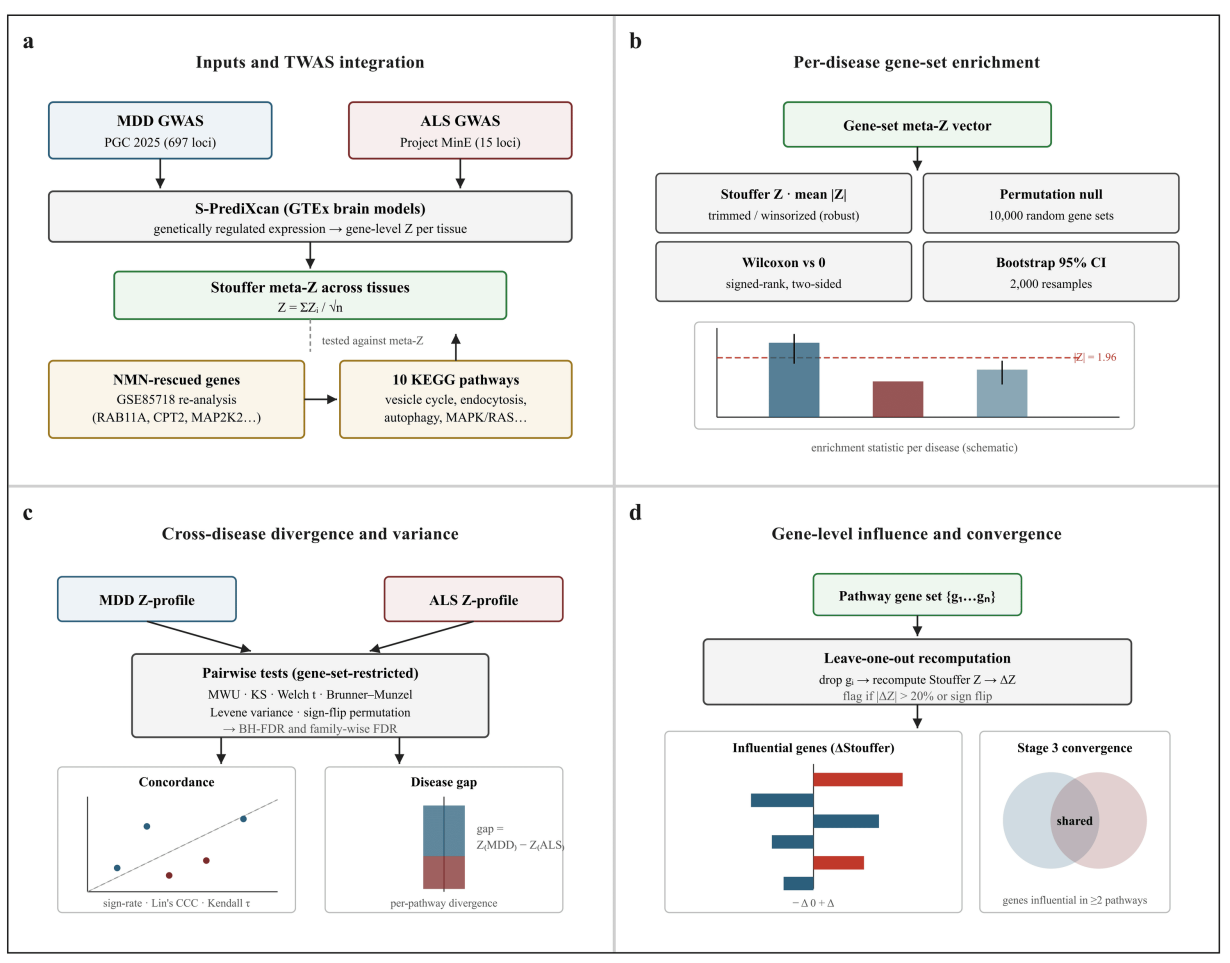
Analytical pipeline (exploratory secondary TWAS/pathway-integration workflow) Ten KEGG pathways were predefined from prior NMN-related analysis and were not discovered de novo from current MDD or ALS TWAS data. Panel a: use of MDD and ALS S-PrediXcan gene-level Z-scores and cross-tissue Stouffer aggregation. Panel b: per-pathway screening with Stouffer Z, mean absolute Z, Wilcoxon signed-rank testing, competitive permutation testing, and percentile bootstrap intervals. Competitive permutation null sampled gene sets of same size without replacement from all genes with finite Z-scores in relevant disease; it therefore tested competitive enrichment relative to available gene background rather than self-contained pathway activity. Panel c: secondary cross-disease comparisons using distribution, variance, concordance, and divergence metrics. Panel d: leave-one-out sensitivity analysis, in which each gene was removed, and pathway Stouffer statistic recomputed. Genes producing sign flip or change exceeding 20% were labeled statistically influential in sensitivity analysis; they were not treated as validated causal drivers. The run generated 20 meta-across-tissue disease-by-pathway screening combinations, 10 pathway-level disease-pair rows, 10 proximity rows, 190 pooled family-wise FDR records, 2,443 leave-one-out gene-disease entries, and 1,788 per-gene records. The main MDD synaptic vesicle result exceeded the Stouffer screening threshold but did not survive meta-level permutation or Wilcoxon testing. The figure is therefore an exploratory workflow and does not imply causal inference or experimentally validated mechanisms. Figure was created using PowerPoint (Microsoft Corporation, Redmond, WA, USA). LOO: leave-one-out sensitivity/influence analysis; CCC: Lin’s concordance correlation coefficient; KEGG: Kyoto Encyclopedia of Genes and Genomes; NMN: nicotinamide mononucleotide; TWAS: transcriptome-wide association study; MDD: major depressive disorder; ALS: amyotrophic lateral sclerosis; BH-FDR: Benjamini-Hochberg false discovery rate

This was an exploratory secondary integrative analysis of public GWAS and precomputed TWAS-derived summary outputs (Figure 1). MDD genetic association data were based on the 2025 trans-ancestry depression GWAS from the Major Depressive Disorder Working Group of the Psychiatric Genomics Consortium, which reported 697 associations and implicated relevant cell types and pharmacotherapies [6]. ALS data were derived from Project MinE and related large-scale ALS GWAS work, including the study by van Rheenen and colleagues, which identified 15 ALS risk loci and highlighted distinct genetic architectures involving rare variants, repeat expansions, regulatory effects, autophagy, vesicle-mediated transport, and neuron-specific biology [5].

The analysis used precomputed S-PrediXcan result files rather than rerunning the upstream GWAS-to-expression prediction models. The source run used brain-relevant tissue files, including amygdala and anterior cingulate cortex BA24 outputs, where available. Genes were retained when finite gene-level Z-scores were available. The exact PredictDB model release, GTEx model version, and complete software-package version manifest were not retained in the run record; this limits exact computational reproducibility and is considered below.

The analysis was informed by the previously published MDD-ALS pruning-continuum model, which used MAGMA, GSEA/DGSEA, S-PrediXcan, and LDSC to show near-zero genome-wide genetic correlation but pathway-level convergence on synaptic pruning biology [4]. The present work did not repeat that full eight-set analysis. Instead, it tested a focused NMN-nominated pathway panel against MDD and ALS TWAS outputs.

Pathway nomination, orthology mapping, and gene-set construction

The pathway panel was predefined before the current MDD/ALS TWAS screen from the author’s prior re-analysis of GSE85718 [7], the dataset underlying the long-term NMN administration study in aged mice [8]. The prior analysis identified 35 genes reported as robustly rescued by NMN in at least two of three peripheral metabolic tissues. The current source record did not preserve a formal over-representation p-value threshold, a preregistered pathway-retention rule, or a complete list of KEGG pathways that were considered and excluded. Accordingly, the 10-pathway panel is treated here as an author-nominated exploratory panel and as a potential source of selection bias, rather than as an independently validated enrichment result.

Mouse-derived candidate genes were converted to human ortholog gene symbols before construction of the human pathway panel using Ensembl/BioMart-compatible orthology resources. The exact orthology database release was not retained in the run manifest. The resulting human gene sets were mapped to KEGG pathway definitions [12]. The nominated pathways were KEGG Endocytosis, KEGG Fatty Acid Degradation, KEGG MAPK Signaling Pathway, KEGG RAS Signaling Pathway, KEGG PPAR Signaling Pathway, KEGG Neuroactive Ligand-Receptor Interaction, KEGG Regulation of Actin Cytoskeleton, KEGG Citrate Cycle/TCA Cycle, KEGG Autophagy-Animal, and KEGG Synaptic Vesicle Cycle.

The strongest mechanistic leads were RAB11A, linked to endosomal recycling and endocytosis; CPT2, linked to mitochondrial long-chain fatty-acid oxidation; MAP2K2, linked to MAPK and RAS signaling; MYO15, linked to actin/cytoskeletal biology; GALR1, linked to neuroactive ligand-receptor signaling; SLC13A5, linked to citrate transport and central carbon metabolism; TRP53INP2, linked to autophagy-related regulation; and APBA2, linked more weakly to synaptic vesicle-associated biology.

Importantly, the current run tested the 10 nominated pathways, not the direct 35-gene NMN-robust list as a standalone gene set. The NMN_ROBUST list used by the Stage 3 configuration was empty, so no formal overlap analysis between the 35 prior genes and the leave-one-out influential genes was performed.

TWAS and enrichment analysis

TWAS integration used S-PrediXcan principles, which infer the phenotypic consequences of genetically regulated tissue-specific expression from GWAS summary statistics [13]. The broader rationale follows the transcriptome-imputation framework in which GWAS signals are mapped to predicted gene expression across tissues, reducing single-variant complexity and enabling gene-level interpretation. Tissue models were based on GTEx-derived prediction resources, and the supplied pipeline prioritized brain-relevant S-PrediXcan outputs where available [14].

For each disease and pathway, the available tissue-level gene Z-scores were combined using the statistic Z_meta = sum of tissue-level Z-scores divided by the square root of the number of available tissue observations. This calculation did not explicitly model cross-tissue correlation. Gene-set coverage was calculated as the proportion of nominated genes with finite Z-scores in the relevant disease or tissue. Missing genes were excluded from the corresponding calculation, and paired disease comparisons used only genes present in both disease datasets.

The multi-gene-set pipeline computed meta-across-tissue enrichment, tissue-specific enrichment, Wilcoxon signed-rank tests where sample size permitted, permutation p-values using 10,000 permutations, bootstrap confidence intervals, and robust winsorized statistics. The completed run used 2,000 bootstrap resamples and robust 1st/99th-percentile winsorization for the requested robust analyses. Tissue-level permutation testing was disabled in the multi-gene-set call, so tissue-level permutation p-values were not available. Enrichment was considered notable when Stouffer Z exceeded an absolute threshold of 1.96, with p-value and FDR information reported separately. Because some signals exceeded the Stouffer threshold without passing permutation or Wilcoxon testing, results are described as exploratory pathway signals unless corrected tests supported stronger claims.

The downstream relevance ranking used the descriptive score:

$0.60 \times \left| Z_{\mathrm{Stouffer}} \right| + 0.25 \times \text{coverage fraction} + 0.15 \times \left(1 + \text{bootstrap confidence interval width}\right)$

When a confidence interval was unavailable, the ranking script used a default width of 10. This composite score was not a statistical test, was not used to establish significance, and should not be interpreted as a probability of enrichment.

Downstream divergence and influence analysis

Downstream analyses examined disease differences within each pathway. Pairwise disease statistics included Mann-Whitney U, Kolmogorov-Smirnov, Welch t, Levene variance tests, Brunner-Munzel tests, paired t-tests, Wilcoxon signed-rank tests, sign-flip permutation tests, Cohen’s d, rank-biserial correlation, sign concordance, Lin’s concordance correlation coefficient, and Kendall tau.

The analysis hierarchy was specified as follows. The meta-across-tissue Stouffer Z values for the 10 pathways in each of the two diseases constituted the primary exploratory screening family. Competitive permutation and Wilcoxon tests were complementary checks on those pathway-level signals, not independent confirmatory analyses. Tissue-level tests, differential tests, proximity measures, variance tests, concordance measures, leave-one-out influence analysis, and Stage 3 pathway-overlap analysis were secondary exploratory analyses.

False-discovery correction was applied within relevant p-value families, and an additional pooled family-wise FDR analysis was computed across the p-values generated within each gene-set run. In the multi-gene-set pairwise output, global FDR values were also calculated separately for each p-value column across the 10 pathway rows. No p-values were generated for the leave-one-out or Stage 3 influence counts; these analyses were descriptive sensitivity analyses rather than formal hypothesis tests.

Leave-one-out influence analysis recalculated Stouffer Z after removing each gene from a pathway. A gene was classified as statistically influential in sensitivity analysis if its removal changed the absolute Stouffer statistic by more than 20% or produced a sign flip. This criterion identifies sensitivity to individual genes; it does not demonstrate causal importance, biological centrality, or a validated driver role.

Stage 3 analysis focused on four automatically selected divergent pathways: Synaptic Vesicle Cycle, RAS Signaling Pathway, Neuroactive Ligand-Receptor Interaction, and Regulation of Actin Cytoskeleton. Convergent genes were defined as genes meeting the leave-one-out influence criterion in at least two focus pathways. The term “convergent” therefore refers to repeated statistical influence across pathway analyses and not to proven convergence of disease mechanisms.

Multiple testing and interpretation framework

The complete testing inventory and interpretation hierarchy are summarized in Table 1. Detailed results for each analysis family, including full gene lists and additional statistical outputs, are provided in Appendices 1-12. The analysis was exploratory at every level. Findings were described as corrected only when the relevant reported FDR criterion was met. Descriptive pathway gaps, concordance values, influence counts, and Stage 3 overlaps were not treated as inferential evidence.

**Table 1:** Statistical testing inventory and interpretation hierarchy BH-FDR: Benjamini-Hochberg false discovery rate; FDR: false discovery rate

Analysis family	Completed assessments	Role	Correction or interpretation	Main status
Meta-across-tissue pathway screening	20 Stouffer evaluations; 20 Wilcoxon checks; 20 competitive permutations	Primary exploratory screen	Stouffer threshold, raw permutation, and Wilcoxon p-values; pooled FDR reported separately	No meta-level pathway passed the permutation/Wilcoxon criteria
Tissue-level enrichment	Included within 100 enrichment records loaded downstream	Secondary exploratory	Tissue-level Wilcoxon and Stouffer screening; tissue permutation testing disabled	Amygdala synaptic vesicle signal had a nominal Wilcoxon p = 0.0093
Differential tests	10 Kruskal-Wallis tests and 10 pairwise differential rows	Secondary exploratory	Within-output BH-FDR	No significant differential findings
Profile proximity	10 disease-pair rows with Pearson and Spearman tests	Secondary descriptive	Correlation p-values included in pooled FDR calculations	No significant proximity pairs
Comprehensive pairwise statistics	10 pathway-level disease-pair rows; up to 100 p-value tests across the reported test families	Secondary exploratory	Column-wise BH-FDR and combined global FDR	Actin cytoskeleton and endocytosis Levene results survived reported global FDR
Pooled family-wise FDR	190 p-value records	Multiple-testing sensitivity analysis	BH-FDR across p-values pooled within each gene-set run	Two reported global FDR survivors
Leave-one-out influence	2,443 gene-disease entries; 1,024 flagged entries	Sensitivity analysis	No p-values or FDR	Descriptive statistical influence only
Stage 3 convergence	14 genes influential in at least two focus pathways	Secondary hypothesis generation	No p-values or FDR	Candidate pathway-overlap signals only

Statistical framework and interpretation

This analysis was designed as hypothesis-generating. It used gene-set and TWAS methods conceptually related to MAGMA (Multi-marker Analysis of GenoMic Annotation) and GSEA (Gene Set Enrichment Analysis) frameworks, but did not apply MAGMA or GSEA to the current data [15,16]. LDSC-based concepts were used for contextual interpretation of polygenicity and pathway-level versus genome-wide sharing [17,18], while the prior MDD-ALS pruning-continuum manuscript was used as conceptual background [4]. No individual-level genotype data, clinical outcome data, colocalization, fine-mapping, Mendelian-randomization analysis, or functional validation experiments were used in the present analysis.

## Results

Overview of pathway-level signals

Across the 10 NMN-nominated KEGG pathways, the clearest meta-across-tissue result was the strongest Stouffer-based exploratory signal, not a statistically confirmed enrichment. KEGG Synaptic Vesicle Cycle ranked first among all gene set-disease combinations, with a composite relevance score of 2.224 and a meta-across-tissue Stouffer Z of 3.41. Coverage for this MDD synaptic vesicle signal was 65%. The bootstrap 95% confidence interval was wide, from −0.46 to 7.40, and the meta-level permutation p-value and Wilcoxon p-value did not pass 0.05 (permutation p = 0.1222; Wilcoxon p = 0.1926). The result should therefore be read as a Stouffer-based exploratory signal that did not survive meta-level permutation or Wilcoxon testing. The 10 NMN-nominated pathways and their leading disease-level combinations are summarized in Table 2.

**Table 2:** Exploratory ranking of NMN-nominated KEGG pathways and leading disease-level signals The table emphasizes exploratory ranking rather than confirmed enrichment. The composite score was calculated as $0.60\,\left|Z_{\mathrm{Stouffer}}\right| + 0.25\,\mathrm{CoverageFraction} + 0.15\left(1 + \mathrm{BootstrapCIWidth}\right)$. The score is descriptive and does not replace permutation, Wilcoxon, or FDR testing. “Meta-level permutation/Wilcoxon/FDR survival” indicates whether the listed disease-level combination passed the reported meta-level checks; no listed combination did so. NMN: nicotinamide mononucleotide; KEGG: Kyoto Encyclopedia of Genes and Genomes; TWAS: transcriptome-wide association study; MDD: major depressive disorder; ALS: amyotrophic lateral sclerosis; Z: Stouffer combined Z-statistic; FDR: false discovery rate

Rank	Gene set	NMN-linked seed / mechanistic lead	Leading disease	Composite score	Z	Coverage	Permutation p	Meta-level permutation/Wilcoxon/FDR survival
1	KEGG_SYNAPTIC_VESICLE_CYCLE	APBA2	MDD	2.224	3.41	65%	0.1222	No
2	KEGG_RAS_SIGNALING_PATHWAY	MAP2K2	MDD	1.225	−1.74	66%	0.4245	No
3	KEGG_AUTOPHAGY_ANIMAL	TRP53INP2	MDD	0.960	−1.26	75%	0.5679	No
4	KEGG_NEUROACTIVE_LIGAND_RECEPTOR_INTERACTION	GALR1	ALS	0.816	1.07	60%	0.5257	No
5	KEGG_MAPK_SIGNALING_PATHWAY	MAP2K2	ALS	0.745	0.93	66%	0.5904	No
6	KEGG_REGULATION_OF_ACTIN_CYTOSKELETON	MYO15	MDD	0.735	0.90	70%	0.6815	No
7	KEGG_FATTY_ACID_DEGRADATION	CPT2	MDD	0.674	−0.77	79%	0.7244	No
8	KEGG_ENDOCYTOSIS	RAB11A	ALS	0.498	0.48	76%	0.7852	No
9	KEGG_CITRATE_CYCLE_TCA_CYCLE	SLC13A5	MDD	0.411	0.31	83%	0.8864	No
10	KEGG_PPAR_SIGNALING_PATHWAY	CPT2	ALS	0.402	−0.35	71%	0.8367	No

The complete gene-set relevance ranking across all disease-pathway combinations is presented in Appendix 2. At the tissue level, however, the MDD synaptic vesicle cycle signal was sharper. The strongest tissue-specific result occurred in the amygdala, with Stouffer Z = 4.057 and Wilcoxon p = 0.0093. This was a nominal tissue-level result; tissue-level permutation testing was not performed in the multi-gene-set run, and a tissue-level FDR survivor was not reported. Additional tissue-level MDD signals included Regulation of Actin Cytoskeleton in amygdala (Z = 2.176), MAPK Signaling Pathway in anterior cingulate cortex BA24 (Z = −2.074), RAS Signaling Pathway in anterior cingulate cortex BA24 (Z = −2.666), and Citrate Cycle/TCA Cycle in amygdala (Z = 2.321). These tissue-specific results fit the clinical expectation that limbic and anterior cingulate circuits are central to affective symptoms, stress sensitivity, and synaptic plasticity [19]. Because the data were derived from bulk-tissue prediction models, the tissue associations do not establish cell-type-specific limbic mechanisms. Exploratory pathway signals are shown in Table 3. The full set of per-disease pathway signals meeting exploratory criteria is listed in Appendix 1.

**Table 3:** Per-disease pathway signals meeting exploratory reporting criteria (|Stouffer Z| threshold or nominal Wilcoxon p < 0.05) The table is not a list of corrected statistically significant enrichment findings. It lists pathway-level signals meeting the exploratory reporting rule. The tissue-level permutation column is NA because tissue-level permutations were disabled in the multi-gene-set run. KEGG: Kyoto Encyclopedia of Genes and Genomes; MDD: major depressive disorder; ALS: amyotrophic lateral sclerosis; TWAS: transcriptome-wide association study; Z: Stouffer statistic; n: number of genes tested; FDR: false discovery rate

Gene set	Disease	Tissue/level	n	Stouffer Z	Permutation p	Wilcoxon p	Exploratory criterion	Correction status
KEGG_SYNAPTIC_VESICLE_CYCLE	MDD	Brain_Amygdala_spredixcan	30	4.057	NA	0.009301		Z
KEGG_REGULATION_OF_ACTIN_CYTOSKELETON	MDD	Brain_Amygdala_spredixcan	108	2.176	NA	0.06951		Z
KEGG_MAPK_SIGNALING_PATHWAY	MDD	Brain_Anterior_cingulate_cortex_BA24_spredixcan	147	−2.074	NA	0.07366		Z
KEGG_RAS_SIGNALING_PATHWAY	MDD	Brain_Anterior_cingulate_cortex_BA24_spredixcan	121	−2.666	NA	0.07409		Z
KEGG_SYNAPTIC_VESICLE_CYCLE	MDD	Meta-across-tissues	51	3.410	0.1222	0.1926		Z
KEGG_CITRATE_CYCLE_TCA_CYCLE	MDD	Brain_Amygdala_spredixcan	21	2.321	NA	0.2029		Z

ALS showed no significant meta-across-tissue pathway enrichment among the 10 NMN-nominated KEGG pathways. This absence of meta-level enrichment is important and should not be obscured by the candidate gene-level findings. ALS signals appeared concentrated in particular genes and processes rather than distributed evenly enough across broad KEGG pathways to generate meta-level enrichment. In the gene-set relevance ranking, the leading ALS pathway combinations were Synaptic Vesicle Cycle (Z = −1.17), Neuroactive Ligand-Receptor Interaction (Z = 1.07), MAPK Signaling Pathway (Z = 0.93), Autophagy-Animal (Z = −0.63), and Endocytosis (Z = 0.48). These values did not meet the same meta-level screening threshold as the MDD Synaptic Vesicle Cycle signal. The candidate ALS gene-level findings are therefore directionally consistent with known autophagy and endosomal biology but do not constitute new evidence that ALS broadly supports the NMN-nominated pathway model.

Synaptic vesicle cycle: the strongest MDD exploratory signal and the largest MDD-ALS divergence

The synaptic vesicle cycle was the most prominent exploratory pathway in the analysis. It was the only meta-level MDD signal exceeding the Stouffer screening threshold and produced the largest descriptive cross-disease gap: MDD Z = 3.41 compared with ALS Z = −1.17, yielding a gap of 4.58. This gap is descriptive and should not be interpreted as a formal disease-divergence test by itself. Concordance metrics indicated little shared gene-level directionality between the two diseases within this pathway. The sign-concordance rate was 0.431, Lin’s concordance correlation coefficient was −0.070, and Kendall's tau was −0.029. In plain terms, the same pathway label was associated with different gene-level patterns in the two diseases.

Focus-pathway divergence and concordance metrics are shown in Table 4, and the largest cross-disease signal gaps are shown in Table 5. The full ranking of cross-disease Stouffer Z gaps is shown in Appendix 4.

**Table 4:** Focus-pathway divergence and concordance metrics Influential entries were defined as genes whose removal changed the absolute Stouffer statistic by more than 20% or produced a sign flip. The counts are sensitivity-analysis entries and may be affected by pathway size and heterogeneity; they are not causal-driver counts. NR indicates that the denominator or exact value was not retained in the reported summary and was not inferred. KEGG: Kyoto Encyclopedia of Genes and Genomes; CCC: Lin’s concordance correlation coefficient; FDR: false discovery rate; LOO: leave-one-out; τ: Kendall tau; MDD: major depressive disorder; ALS: amyotrophic lateral sclerosis

Focus pathway	ALS Z	MDD Z	Gap	n paired genes	Levene result (raw p; FDR)	Influential entries (LOO criterion)	Sign concordance	CCC	τ	Concordance criteria met
KEGG_SYNAPTIC_VESICLE_CYCLE	−1.17	3.41	4.58	NR	No; raw p/FDR NR	25	0.431	−0.070	−0.029	No
KEGG_RAS_SIGNALING_PATHWAY	0.24	−1.74	1.98	NR	Yes; exact raw p/FDR NR	48	0.460	−0.008	0.004	No
KEGG_NEUROACTIVE_LIGAND_RECEPTOR_INTERACTION	1.07	−0.24	1.31	218	Yes; raw p NR; FDR 0.009707	190	0.514	−0.062	−0.004	No
KEGG_REGULATION_OF_ACTIN_CYTOSKELETON	−0.14	0.90	1.04	151	Yes; raw p 0.001659; FDR 0.008495	123	0.443	0.016	0.000	No

**Table 5:** Descriptive ranking of cross-disease Stouffer signal gaps The gap is defined as the absolute difference between the disease-specific Stouffer Z values. It is a descriptive ranking and not, by itself, a formal statistical test of disease divergence. Paired disease tests and FDR results are reported separately where available. KEGG: Kyoto Encyclopedia of Genes and Genomes; MDD: major depressive disorder; ALS: amyotrophic lateral sclerosis; FDR: false discovery rate

Rank	Gene set	Gap	ALS Z	MDD Z	Paired-gene denominator	Interpretation
1	KEGG_SYNAPTIC_VESICLE_CYCLE	4.58	−1.17	3.41	NR	Descriptive gap; no corrected gap test reported
2	KEGG_RAS_SIGNALING_PATHWAY	1.98	0.24	−1.74	NR	Descriptive gap; no corrected gap test reported
3	KEGG_NEUROACTIVE_LIGAND_RECEPTOR_INTERACTION	1.31	1.07	−0.24	NR	Descriptive gap; no corrected gap test reported
4	KEGG_REGULATION_OF_ACTIN_CYTOSKELETON	1.04	−0.14	0.90	NR	Descriptive gap; no corrected gap test reported
5	KEGG_MAPK_SIGNALING_PATHWAY	1.03	0.93	−0.10	NR	Descriptive gap; no corrected gap test reported
6	KEGG_FATTY_ACID_DEGRADATION	0.99	0.22	−0.77	NR	Descriptive gap; no corrected gap test reported
7	KEGG_AUTOPHAGY_ANIMAL	0.63	−0.63	−1.26	NR	Descriptive gap; no corrected gap test reported
8	KEGG_PPAR_SIGNALING_PATHWAY	0.55	−0.35	0.20	NR	Descriptive gap; no corrected gap test reported
9	KEGG_CITRATE_CYCLE_TCA_CYCLE	0.44	−0.13	0.31	NR	Descriptive gap; no corrected gap test reported
10	KEGG_ENDOCYTOSIS	0.09	0.48	0.39	NR	Descriptive gap; no corrected gap test reported

The most striking individual gene was VAMP2. In MDD, VAMP2 showed Z = 7.97; in ALS, it showed Z = −5.32. The resulting divergence was 13.29, the largest gene-level divergence observed in the synaptic vesicle pathway. VAMP2 encodes a core vesicle-associated SNARE protein involved in synaptic vesicle fusion and neurotransmitter release. Its opposite-direction profile across MDD and ALS is therefore biologically provocative but should be interpreted cautiously. Opposite TWAS Z-scores do not necessarily represent opposite disease biology. They may reflect differences in tissue composition, expression-prediction models, linkage disequilibrium structure, ancestry, GWAS power, or model stability. Without colocalization, fine-mapping, and functional validation, VAMP2 should be regarded as a candidate signal rather than evidence of disease-specific vesicle dysfunction.

Several additional synaptic vesicle genes contributed to the divergence. In ALS, ATP6V0D1 was strongly positive (Z = 7.64), while ATP6V1D (Z = −4.52), ATP6V0A1 (Z = −3.53), ATP6V1E2 (Z = −3.17), DNM3 (Z = −3.05), NAPA (Z = −2.80), STX1A (Z = −2.05), and STX1B (Z = −1.73) were influential. In MDD, ATP6V0B showed a strong positive signal (Z = 5.39), STX3 was positive (Z = 3.79), and DNM1 was positive (Z = 3.49), despite DNM1 being negative in ALS (ALS Z = −1.39; divergence = 4.88). Clathrin-related genes also diverged; CLTCL1 showed MDD Z = −3.26 and ALS Z = 1.39, while CLTA was positive in ALS (Z = 2.41) and near neutral in MDD.

The biological theme is coherent. Vesicle acidification, clathrin-mediated recycling, dynamin-dependent endocytosis, SNARE-mediated fusion, and syntaxin function all belong to the machinery that determines whether synapses can release neurotransmitter, recycle vesicles, and maintain presynaptic stability. These processes are also intimately linked to synaptic vesicle endocytosis and turnover [20,21]. The present findings suggest that synaptic logistics are a candidate area for further investigation, but they do not establish that intrinsic presynaptic machinery is causally responsible for MDD-ALS overlap or for microglial pruning.

Variance heterogeneity in pathways relevant to cellular logistics

Mean pathway differences were not the main cross-disease finding. Instead, exploratory variance heterogeneity stood out. Pairwise testing identified Levene variance differences between MDD and ALS in Regulation of Actin Cytoskeleton, Endocytosis, and Neuroactive Ligand-Receptor Interaction. In the initial pairwise analysis, Regulation of Actin Cytoskeleton had Levene FDR = 0.0085, Endocytosis had Levene FDR = 0.0085, and Neuroactive Ligand-Receptor Interaction had Levene FDR = 0.0097. When the reported in-run p-values were pooled into the multi-gene-set global FDR calculation, Regulation of Actin Cytoskeleton and Endocytosis remained significant, with global FDR values of 0.0315 and 0.0323, respectively. These results are exploratory evidence of distributional heterogeneity rather than proof of distinct disease mechanisms (Table 6, 7). Full pairwise disease statistical test results, including those surviving global FDR correction, are detailed in Appendices 5, 6.

**Table 6:** Exploratory pairwise variance comparisons KEGG: Kyoto Encyclopedia of Genes and Genomes; MDD: major depressive disorder; ALS: amyotrophic lateral sclerosis; FDR: false discovery rate

Gene set	Contrast	n paired	Mean difference	Cohen d paired	Cohen d unpaired	Levene raw p	Per-run FDR	Combined global FDR	Interpretation
KEGG_REGULATION_OF_ACTIN_CYTOSKELETON	MDD vs ALS / Levene	151	0.059	0.01	0.05	0.001659	0.008495	0.03152	Exploratory variance heterogeneity; survived reported global FDR
KEGG_ENDOCYTOSIS	MDD vs ALS / Levene	186	0.054	0.02	−0.00	0.001699	0.008495	0.03228	Exploratory variance heterogeneity; survived reported global FDR
KEGG_NEUROACTIVE_LIGAND_RECEPTOR_INTERACTION	MDD vs ALS / Levene	218	−0.102	−0.05	−0.06	NR	0.009707	NR	Per-run FDR reported; global FDR survival not reported

**Table 7:** Tests surviving the reported combined global FDR analysis Raw p-values are shown where retained in the reported output. NR indicates that the raw p-value was not retained in the available summary. Per-run FDR refers to the FDR reported within the corresponding pathway-level run; combined global FDR refers to the multi-gene-set pairwise calculation across the 10 pathway rows for the relevant p-value column. KEGG: Kyoto Encyclopedia of Genes and Genomes; FDR: false discovery rate

Gene set	Test	n paired	Raw p	Per-run FDR	Combined global FDR	Status
KEGG_REGULATION_OF_ACTIN_CYTOSKELETON	Levene variance test	151	0.001659	0.008495	0.03152	Survived reported global FDR < 0.05
KEGG_ENDOCYTOSIS	Levene variance test	186	0.001699	0.008495	0.03228	Survived reported global FDR < 0.05

This matters because variance differences can reveal biology that mean differences miss. Endocytosis and the actin cytoskeleton are core logistics pathways for microglial process movement, receptor internalization, synapse engulfment, vesicle recycling, and membrane remodeling. Actin dynamics regulate the physical extension and retraction of microglial processes and shape neuronal spine structure. Endosomal pathways regulate receptor recycling and vesicle sorting. These processes are relevant to microglial pruning and neuronal synaptic maintenance [9,22,23]. Because the present data derive from bulk-tissue TWAS outputs, the reference to microglial process movement and neuronal spine structure is inferential and requires validation in cell-type-specific or single-cell-informed models.

The gene-level pattern was disease-specific. In ALS endocytosis, influential genes included RAB5C (Z = 5.13), ARFGAP2 (Z = −4.59), VPS45 (Z = −4.38), RAB8A (Z = −3.76), RAB5B (Z = 3.50), RAB7A (Z = −3.13), DNM3 (Z = −3.05), WASL (Z = 2.87), ARPC5 (Z = 2.71), SNX3 (Z = −2.56), and CHMP2B (Z = 1.88). In MDD endocytosis, influential genes included PML (Z = 7.19), VPS45 (Z = −6.46), SNF8 (Z = 6.41), ARFGEF2 (Z = −5.87), PSD (Z = 5.06), IST1 (Z = −4.97), RAB10 (Z = 4.24), HLA-C (Z = −4.08), DNM1 (Z = 3.49), and many ESCRT, clathrin, ARF, RAB, and sorting nexin genes. The overlap was not absent, but the directional pattern was inconsistent.

In actin cytoskeleton, ALS influential genes included EGF, KNG1, ITGA3, LIMK2, WASL, ARPC5, ITGA1, FN1, ARPC2, ITGB3, ROCK1, RAC1, PFN1, MAPK1, PAK1, and PAK2. MDD influential genes included PPP1CB (Z = 8.70), DIAPH3, FN1, NCKAP1, ITGB4, RRAS2, PTK2, GNA12, RAC3, KNG1, EGF, ARHGEF12, GSN, CFL2, FGF8, FGFR4, ACTG1, AKT3, and ARPC1A. These lists identify statistical sensitivity to individual genes within the pathway calculations; they do not establish that the listed genes are shared biological drivers.

Candidate statistically influential genes at the RAS-actin-MAPK interface

Stage 3 convergence analysis identified 14 genes that met the leave-one-out influence criterion in at least two focus pathways: EGF, KNG1, FGF8, FGF17, RAC1, FGFR1, MAPK1, BDKRB1, PIK3R3, PAK2, RAF1, PAK1, BDKRB2, and CHRM3 (Table 8). The complete list of genes meeting the leave-one-out influence criterion across focus pathways is provided in Appendix 9; the broader set of most influential genes across all gene sets appears in Appendix 8. Most appeared at the intersection of RAS signaling and actin cytoskeleton regulation. This is biologically plausible because growth-factor signaling, MAPK cascades, actin remodeling, synaptic structure, and immune-cell motility are coupled processes. However, these genes should be regarded as candidate statistically influential genes from sensitivity analysis, not as convergent driver nodes or validated therapeutic targets.

**Table 8:** Candidate genes statistically influential across at least two focus pathways The influence criterion was removal-induced change in absolute Stouffer Z greater than 20% or a sign flip. Disease-specific influence flags and exact Z-scores were not retained for every entry in the reported summary; NR values were not inferred. The listed genes, therefore, represent candidate sensitivity signals rather than causal or validated shared nodes. |Δ|: mean absolute change in Stouffer Z; MDD: major depressive disorder; ALS: amyotrophic lateral sclerosis; NR: not retained in the reported summary

Rank	Gene	No. of pathways	Focus pathways	Mean |Δ|	Sign flip	MDD gene Z	ALS gene Z	Disease-specific influence status
1	EGF	2	RAS signaling; regulation of actin cytoskeleton	0.399	Yes	-3.46	-5.73	NR
2	KNG1	2	Neuroactive ligand–receptor interaction; regulation of actin cytoskeleton	0.293	Yes	-3.75	4.25	NR
3	FGF8	2	RAS signaling; regulation of actin cytoskeleton	0.229	No	-3.31	2.65	NR
4	FGF17	2	RAS signaling; regulation of actin cytoskeleton	0.155	No	-1.75	1.94	NR
5	RAC1	2	RAS signaling; regulation of actin cytoskeleton	0.151	No	0.31	-1.88	NR
6	FGFR1	2	RAS signaling; regulation of actin cytoskeleton	0.143	No	NR	NR	NR
7	MAPK1	2	RAS signaling; regulation of actin cytoskeleton	0.142	No	NR	NR	NR
8	BDKRB1	2	Neuroactive ligand–receptor interaction; regulation of actin cytoskeleton	0.142	Yes	NR	NR	NR
9	PIK3R3	2	RAS signaling; regulation of actin cytoskeleton	0.141	No	NR	NR	NR
10	PAK2	2	RAS signaling; regulation of actin cytoskeleton	0.127	No	NR	NR	NR
11	RAF1	2	RAS signaling; regulation of actin cytoskeleton	0.125	No	NR	NR	NR
12	PAK1	2	RAS signaling; regulation of actin cytoskeleton	0.124	No	NR	NR	NR
13	BDKRB2	2	Neuroactive ligand–receptor interaction; regulation of actin cytoskeleton	0.096	No	NR	NR	NR
14	CHRM3	2	Neuroactive ligand–receptor interaction; regulation of actin cytoskeleton	0.062	No	NR	NR	NR

EGF was influential in both RAS signaling and the regulation of the actin cytoskeleton and showed sign-flip behavior. KNG1 appeared in Neuroactive Ligand-Receptor Interaction and Regulation of Actin Cytoskeleton, also with sign-flip behavior. FGF8 and FGF17 bridged RAS and actin pathways. RAC1, PAK1, PAK2, RAF1, MAPK1, and FGFR1 form a coherent signaling module linking membrane receptor activation to cytoskeletal remodeling and downstream kinase signaling. MAPK signaling is also implicated in stress responses, synaptic plasticity, and antidepressant-related mechanisms [19]. Per-pathway influential genes identified by leave-one-out analysis for the four focus pathways are listed in Appendix 10.

These genes do not prove shared causality. They provide a compact set of candidate signals for replication, colocalization, fine-mapping, and functional testing. In MDD, chronic stress, immune signaling, and synaptic remodeling could place pressure on this RAS-actin-MAPK module in limbic circuits. In ALS, autophagy and endosomal trafficking stress could affect overlapping machinery in motor or glutamatergic neurons. Such interpretations remain inferential because the current analysis used bulk-tissue genetically regulated expression rather than cell-type-specific measurements.

ALS gene-level coherence with known autophagy and vesicle biology

Although ALS lacked meta-level enrichment across the 10 NMN-nominated KEGG pathways, its candidate gene-level pattern was directionally consistent with established ALS biology. In KEGG Autophagy-Animal, the strongest influential ALS genes were TBK1 (Z = 11.23), C9orf72 (Z = 8.43), EIF2AK3 (Z = 6.60), WIPI2 (Z = −6.20), ATG10 (Z = −5.05), CTSB (Z = −4.80), PIK3R4 (Z = 4.26), RAB8A (Z = −3.76), BCL2L1 (Z = −3.75), ATG4C (Z = 3.68), NBR1 (Z = −3.31), WDR41 (Z = −3.28), RAB7A (Z = −3.13), UVRAG (Z = 3.05), EIF2S1 (Z = 3.00), ATG7 (Z = −2.94), ATG13 (Z = 2.69), TAX1BP1 (Z = −2.10), VPS39 (Z = −2.04), and MAP1LC3B (Z = −2.02). OPTN was also flagged, although with a smaller Z-score in this pathway run.

This pattern is consistent with ALS genetics. TBK1, C9orf72, OPTN, SQSTM1-related pathways, autophagy, endosomal trafficking, lysosomal function, and protein aggregation are central to many models of ALS pathogenesis [24,5]. van Rheenen and colleagues found that ALS risk loci converge on vesicle-mediated transport, Golgi-to-endoplasmic-reticulum trafficking, macroautophagy, and glutamatergic neuron biology. The present analysis is compatible with those themes at the candidate-gene level, but it does not demonstrate a new ALS pathway enrichment or establish that the NMN-nominated panel captures ALS biology broadly.

This difference between pathway-level enrichment and gene-level coherence is important. ALS risk may be concentrated in specific high-impact genes and rare-variant-sensitive processes that are not well captured by broad pathway averaging. MDD, in contrast, may produce broader polygenic shifts across synaptic and signaling pathways. This remains one possible explanation for the observed pattern and requires replication in independent datasets and cell-type-informed analyses.

CPT2 and the metabolic layer

The NMN re-analysis highlighted CPT2 as the only robust mitochondrial gene among the 35 cross-tissue rescued genes [7]. CPT2 is central to mitochondrial long-chain fatty-acid oxidation and connects to fatty-acid degradation and PPAR signaling [25,26]. In the present pathway analysis, fatty-acid degradation and PPAR signaling did not emerge as top meta-level disease enrichments, but several metabolic genes were repeatedly influential.

In ALS fatty-acid degradation, ACSL5 was influential (Z = 4.56), along with ACAT1, ALDH3A2, ALDH2, ADH1C, CPT1C, ACSL1, CPT1B, ACADVL, ACOX1, ACSL6, HADHB, HADH, ECI2, ACAT2, CPT2, CPT1A, and others. In MDD fatty-acid degradation, GCDH, ALDH7A1, ACSL5, ADH5, ACADSB, ACAT2, ACADS, CYP2U1, ACOX1, ADH1C, ALDH2, ACSL1, CPT2, and ALDH3A2 were among influential genes. In PPAR signaling, ALS influential genes included ILK, SCP2, CYP27A1, ACSL5, PPARD, FABP3, DBI, PCK2, NR1H3, FABP1, CPT1C, ACSL1, and CPT1B. MDD influential genes included NR1H3, SLC27A5, PPARD, HMGCS1, APOA2, PLIN5, SLC27A6, ACSL5, UBC, AQP7, PLTP, ACOX1, FABP6, and CPT2.

The metabolic layer, therefore, appeared weaker than the synaptic vesicle or autophagy layers but remained directionally informative. It is compatible with a metabolic-flexibility hypothesis linking substrate handling, lipid metabolism, and autophagy, but it does not establish that NMN or NAD+ repletion would alter these pathways in human MDD or ALS brain tissue. This is relevant to ALS because van Rheenen and colleagues reported Mendelian-randomization evidence consistent with a causal relationship between higher total cholesterol levels and ALS risk and discussed lipid metabolism in relation to autophagy [5]. It is also relevant to MDD, where metabolic stress and treatment resistance often travel together clinically.

Summary of divergence versus convergence

The overall pattern was not one of simple shared directionality. No significant profile-proximity pairs were found. No shared directional programs or opposed programs passed the concordance thresholds. Sign-concordance rates in the focus pathways were low to modest: 0.431 for Synaptic Vesicle Cycle, 0.460 for RAS Signaling Pathway, 0.514 for Neuroactive Ligand-Receptor Interaction, and 0.443 for Regulation of Actin Cytoskeleton. Lin’s concordance coefficients and Kendall tau values were near zero.

This pattern is compatible with a model in which MDD and ALS may affect overlapping cellular-logistics processes while engaging different genes within those processes. It does not establish that the diseases share a single mechanistic layer, and it cannot explain the absence of direct genome-wide genetic correlation on its own.

## Discussion

Mechanistic synthesis

**Figure 2:**
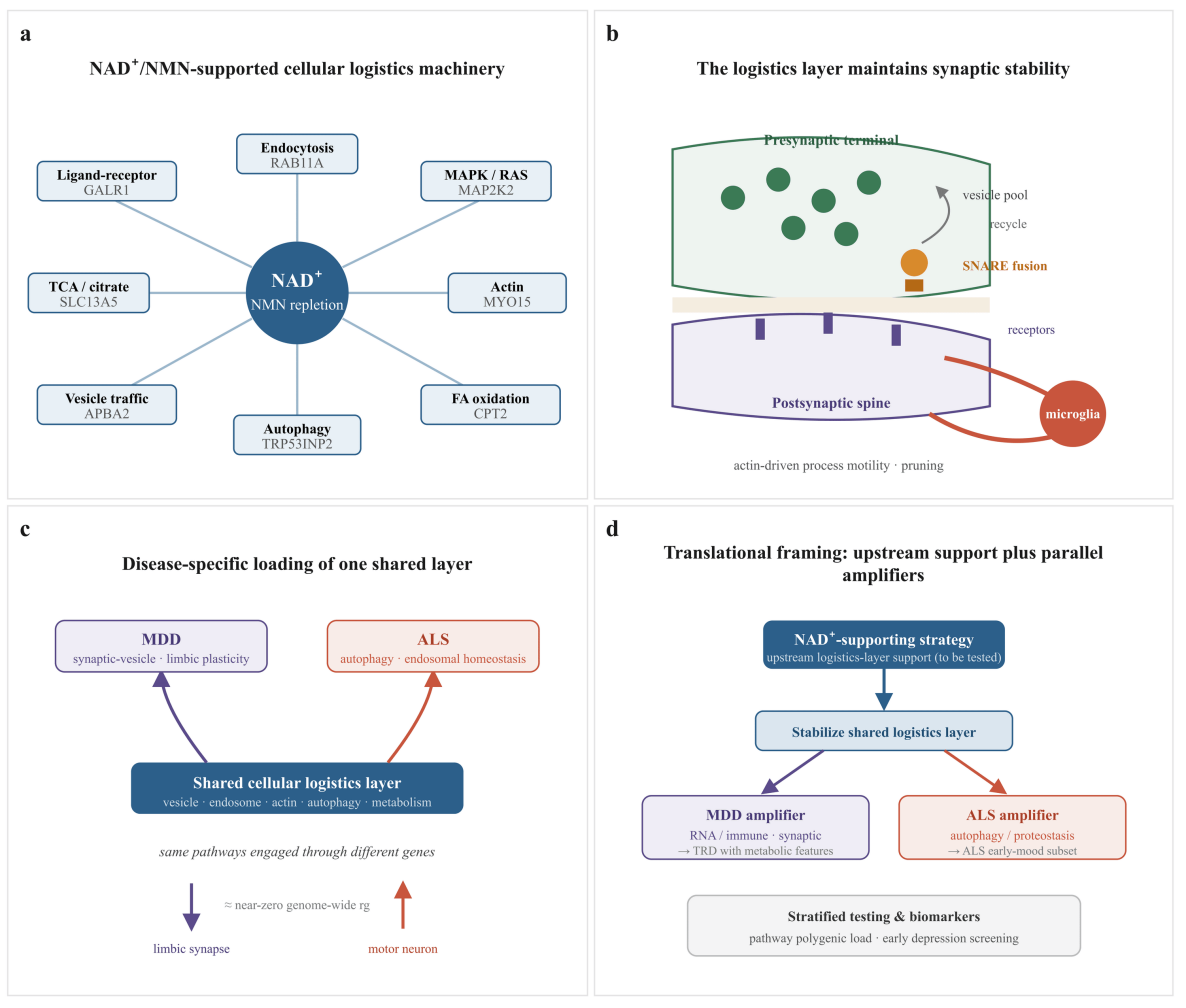
NMN-rescuable cellular logistics as a shared vulnerable layer in MDD–ALS comorbidity This figure presents a hypothesis model derived from exploratory TWAS/pathway integration and should not be interpreted as evidence that NMN treats MDD, ALS, or MDD–ALS comorbidity. Panel a: NMN-associated transcriptional programs identified in prior peripheral metabolic mouse tissues and their possible relationship to candidate processes, including endosomal recycling, vesicle trafficking, actin remodeling, MAPK/RAS signaling, autophagy, and mitochondrial substrate flexibility. Panel b: how these processes may participate in synaptic vesicle handling, receptor turnover, membrane remodeling, and microglial phagocytosis; these cell-type and synapse-specific links are inferential in the present bulk-tissue analysis. Panel c: labels the disease findings cautiously: MDD, the strongest exploratory signal in the synaptic vesicle pathway, especially in the amygdala; and ALS, candidate gene-level coherence in autophagy and endosomal genes without significant meta-pathway enrichment. Panel d: presents future research logic only: NAD+-linked cellular stress pathways may be examined in independent datasets, cell-type-specific models, and functional experiments. The figure does not imply treatment readiness, direct human brain rescue by NMN, or a basis for immediate stratified clinical trials. Figure was created using PowerPoint (Microsoft Corporation, Redmond, WA, USA). NMN: nicotinamide mononucleotide; MDD: major depressive disorder; ALS: amyotrophic lateral sclerosis

The main finding is not that MDD and ALS are genetically similar diseases. They are not. The more cautious interpretation is that the two disorders may place stress on overlapping cellular-logistics processes while loading those processes through different candidate genes. In this NMN-nominated panel, MDD showed the strongest exploratory pathway-level signal in synaptic vesicle biology, particularly in the amygdala. ALS showed candidate gene-level coherence in autophagy and endosomal processes without significant meta-across-tissue pathway enrichment. The pathway names overlap, but the gene-level behavior diverges (Figure 2).

This result refines the pruning-continuum model. The earlier model emphasized shared microglial pruning liability, with ALS amplified by autophagy collapse and MDD amplified by RNA and immune dysregulation [4]. The present analysis adds a more granular hypothesis. Synapses are not just passive targets of pruning. They have intrinsic logistics machinery: vesicle fusion, vesicle acidification, endocytosis, clathrin recycling, dynamin-dependent membrane retrieval, receptor trafficking, cytoskeletal stabilization, and metabolic support. If these processes are fragile, they could influence how synapses respond to stress or pruning; however, this proposed relationship has not been demonstrated by the present TWAS analysis.

This interpretation is consistent with the broader literature. Microglia shape synaptic circuits through complement-dependent mechanisms and phagocytic pruning [27,23]. Patient-derived models show that altered microglial pruning can affect psychiatric risk biology [28]. Depression is strongly linked to synaptic plasticity, stress-related dendritic remodeling, and altered glutamatergic and neurotrophic signaling [19]. ALS genetics, meanwhile, repeatedly points to autophagy, vesicle trafficking, RNA metabolism, and protein aggregation [24,5]. The present results sit at the intersection of these literatures as a hypothesis-generating computational observation, not as functional confirmation.

The variance heterogeneity findings sharpen the model further. Endocytosis and the actin cytoskeleton did not simply shift up or down between MDD and ALS. Their variance differed. This may indicate that the two disorders recruit different gene subsets within related processes, although variance differences in bulk-tissue TWAS data can also arise from differences in tissue composition, prediction-model performance, or GWAS architecture. ALS may stress core endolysosomal and autophagy-linked trafficking. MDD may stress synaptic remodeling, receptor signaling, and cytoskeletal plasticity in limbic circuits. These possibilities require testing in independent and cell-type-informed datasets.

Link to NMN biology

The translational bridge to NMN biology is indirect. The prior NMN findings came from aged mouse metabolic tissues, whereas the current results came from genetically regulated expression associations in human brain-relevant TWAS outputs. The overlap between these domains is therefore a cross-study biological analogy, not evidence of direct pathway rescue.

RAB11A is central here. As a regulator of recycling-endosome organization, RAB11A provides a literal mechanistic link to cellular logistics: recycling-endosome networks influence the return of receptors and membrane proteins to the cell surface and support membrane homeostasis [9,10]. In neurons and immune cells, related recycling machinery could plausibly affect synaptic receptor turnover, vesicle handling, and membrane remodeling, but this inference was not tested directly here. In metabolic tissues, this may relate to insulin receptor trafficking, nutrient transporter dynamics, and mitochondrial quality-control cross-talk. In brain cells, analogous recycling machinery is essential for synaptic receptor turnover, vesicle handling, and microglial membrane remodeling.

CPT2 provides the second major bridge. It links NMN response to fatty-acid oxidation and mitochondrial substrate use. While CPT2 did not dominate the present brain TWAS results, fatty-acid degradation and PPAR-related genes were repeatedly influential, including ACSL5, ACOX1, CPT1B/C, CPT2, PPARD, NR1H3, and lipid-handling genes. This supports a metabolic-flexibility hypothesis rather than a single-gene therapeutic mechanism. NAD+ repletion may eventually be relevant to cellular resilience, energy handling, trafficking, and stress adaptation, but the current data do not show that NMN rescues these pathways in MDD or ALS brain tissue [7-9,11].

Clinical interpretation of depression in ALS

The clinical interpretation of depression in ALS requires particular caution. A patient with ALS may meet criteria for MDD, but may instead have depressive symptoms secondary to disability, fatigue, loss of autonomy, sleep disruption, respiratory compromise, medication effects, or social isolation. Apathy, pseudobulbar affect, and frontotemporal involvement can also resemble or complicate depression. These presentations may coexist, and they cannot be separated using the present genetic pathway analysis.

The possibility of a shared affective vulnerability should therefore be treated as one hypothesis among several. The current findings do not show that early depression is a prodrome of ALS, nor do they show that all mood symptoms in ALS reflect synaptic pruning or metabolic stress. The appropriate clinical implication is early screening, careful syndromal assessment, and longitudinal monitoring of mood, cognition, fatigue, sleep, respiratory status, and motor progression.

Clinical and translational implications

The clinical implication is not that NMN should be prescribed for MDD, ALS, or MDD-ALS comorbidity. No clinical NMN data in these disorders were analyzed, and no biomarker-defined subgroup was tested. The safer interpretation is that these findings generate hypotheses about NAD+-linked cellular stress pathways that may be examined in future preclinical and clinical studies.

A future study could focus on ALS patients with early depressive symptoms, high pruning-related polygenic burden, or autophagy/endosomal-risk profiles. Outcomes would need to include both motor and mood measures, because a therapy that stabilizes synapses may not affect all clinical domains equally. Similarly, in MDD, NAD+-linked strategies might eventually be examined in treatment-resistant subgroups with metabolic dysfunction, inflammatory features, fatigue, cognitive slowing, or biological evidence of impaired plasticity. Such stratification is a future research design, not a therapeutic recommendation supported by the present data.

The gene-level findings also suggest candidate biomarkers. VAMP2, TBK1, C9orf72, CPT2, ACSL5, ACOX1, EGF, RAC1, PAK1/2, MAPK1, RAF1, FGFR1, and FGF8 may be useful as candidate research signals for replication and mechanistic study. They are not ready for clinical use as biomarkers, and the present analysis does not establish that they are causal or disease-specific.

The findings also caution against overly simple interventions. The synaptic vesicle cycle showed opposite-direction gene-level behavior between MDD and ALS, especially VAMP2. A vesicle-related signal could reflect different tissue, cell-type, ancestry, linkage-disequilibrium, or prediction-model contexts rather than opposite biological effects. Functional assays and colocalization analyses are needed before inferring disease-specific vesicle dysfunction or selecting a vesicle-related intervention.

The broader care implication is multidisciplinary. Mood symptoms in ALS should not be treated as psychologically secondary by default. They should be screened early and followed longitudinally. Conversely, treatment-resistant depression with neurological signs, prominent fatigue, metabolic dysfunction, or atypical progression may deserve closer neurological and biological assessment. The value of this work is not that it offers a ready therapy, but that it turns a difficult clinical overlap into a testable biological question.

Limitations and caveats

Several limitations should be kept in view. First, this was an exploratory secondary analysis, and no result was predeclared as confirmatory. The strongest MDD synaptic vesicle result exceeded the Stouffer screening threshold but did not survive meta-level permutation or Wilcoxon testing. The amygdala result was a nominal tissue-level Wilcoxon finding, and tissue-level permutation testing was not performed.

Second, the pathway panel was author-nominated from a prior re-analysis of NMN-associated transcriptional programs. The available pathway-nomination record did not specify a formal enrichment threshold, preregistration, or a complete list of considered and excluded pathways. The 10 pathways may therefore reflect investigator-driven biological selection and require independent validation.

Third, the current analysis tested pathways nominated from a peripheral/metabolic mouse NMN dataset, whereas the disease data were human brain-relevant TWAS outputs. The cross-species and cross-tissue relationship is indirect. The analysis did not demonstrate that NMN rescues any human MDD or ALS brain pathway.

Fourth, TWAS is associational. S-PrediXcan can implicate genetically regulated expression, but it does not prove that altered expression causes disease or that changing expression will improve outcomes [13]. Linkage disequilibrium can produce TWAS associations in the absence of causal mediation. Colocalization, fine-mapping, Mendelian randomization where appropriate, and functional assays are needed. Candidate genes such as VAMP2, TBK1, C9orf72, RAC1, and MAPK1 should therefore not be described as causal drivers.

Fifth, the analysis relied on bulk-tissue S-PrediXcan outputs. Claims involving microglia, motor neurons, glutamatergic neurons, limbic circuits, synaptic pruning, or cell-specific vesicle dysfunction are inferential. Cell-type-specific TWAS, single-cell-informed models, and experimental systems are needed to resolve these mechanisms.

Sixth, the multiple-testing burden was substantial. The run included 10 pathways, two diseases, tissue-level outputs, meta-level enrichment, Wilcoxon tests, competitive permutations, bootstrap intervals, differential tests, proximity correlations, variance tests, concordance measures, leave-one-out analyses, and Stage 3 overlap analyses. Only selected p-value families were FDR-corrected, and leave-one-out and Stage 3 results had no formal p-values. The findings should therefore be interpreted as exploratory, even when individual FDR values were below 0.05.

Seventh, the study did not analyze a participant-level cohort with both MDD and ALS. The phrase "MDD-ALS comorbidity" refers to a cross-disorder conceptual comparison, not observed comorbidity within a single clinical sample. No clinical depression phenotype, longitudinal mood measure, treatment response, imaging marker, or motor outcome was available.

Eighth, the exact PredictDB model release, orthology release, and complete software-package version manifest were not retained in the original run. The source code and run parameters document the statistical logic, but exact model-level reproducibility remains incomplete until the input identifiers, model versions, software environment, and derived files are deposited together.

Future directions

The next step is direct testing of the 35 NMN-robust genes in independent MDD and ALS TWAS outputs, including RAB11A, CPT2, MAP2K2, MYO15, GALR1, SLC13A5, TRP53INP2, and APBA2. Colocalization and fine-mapping approaches such as COLOC, eCAVIAR, FOCUS, and SMR/HEIDI should be used to evaluate whether candidate TWAS signals share causal variants with the underlying disease associations. Cell-type-specific TWAS or single-cell-informed models should be used where possible, especially for motor neurons, glutamatergic neurons, astrocytes, oligodendrocytes, and microglia.

Functional work should test whether NAD+ precursor exposure modifies vesicle trafficking, microglial pruning, endosomal recycling, or autophagy under combined stress conditions. Preclinical models combining chronic stress, pruning activation, metabolic load, and autophagy impairment would be especially informative. Clinically, prospective ALS cohorts should track syndromal depression, apathy, pseudobulbar symptoms, cognition, fatigue, sleep, respiratory status, motor progression, metabolic markers, and genetic pathway burden from the earliest stages.

## Conclusions

This exploratory analysis supports a cautious refinement of the proposed MDD-ALS pruning-continuum model. The two disorders do not appear to share broad genome-wide genetic architecture, but they may affect overlapping cellular-logistics processes that support synaptic and cellular stability. These processes include synaptic vesicle cycling, endocytosis, actin remodeling, MAPK/RAS signaling, autophagy, and metabolic flexibility. MDD showed the strongest exploratory pathway-level signal in synaptic vesicle biology, with the sharpest tissue-level result in the amygdala. ALS showed candidate gene-level coherence in autophagy and endosomal processes, with genes such as TBK1 and C9orf72 aligning with established ALS biology but without significant meta-across-tissue pathway enrichment.

The NMN connection is not a treatment claim. It is an indirect mechanistic clue derived from prior peripheral metabolic mouse data and compared with human brain-relevant TWAS outputs. NMN/NAD⁺ repletion is not established as a treatment for MDD, ALS, or their comorbidity. The present findings instead generate hypotheses about NAD+-linked cellular stress pathways that may be examined in future preclinical and clinical studies. Any such work would require disease-specific calibration because synaptic vesicle genes showed substantial MDD-ALS divergence rather than simple shared directionality. The most useful contribution of this work is therefore conceptual and hypothesis-generating. It moves the proposed pruning-continuum model from a broad pathway hypothesis toward a candidate cellular-process framework. It suggests why early mood symptoms in ALS may warrant biological as well as psychiatric evaluation, while recognizing that depression in ALS is multifactorial and not necessarily shared biology prodromal. It also identifies candidate signals for replication in cell-type-specific, colocalization, fine-mapping, functional, and longitudinal clinical studies.

## Appendices

Appendix 1

**Table 9:** Significant per-disease enrichment findings reported by the summary pipeline Note. The meta-across-tissue synaptic vesicle cycle result had bootstrap CI95 = (−0.46, 7.40), coverage = 65%.

Gene set	Disease	Tissue / level	n	Stouffer Z	Permutation p	Wilcoxon p	Criterion met
KEGG_SYNAPTIC_VESICLE_CYCLE	MDD	Brain_Amygdala_spredixcan	30	4.057	NA	0.009301	Wilcoxon p; |Z|
KEGG_REGULATION_OF_ACTIN_CYTOSKELETON	MDD	Brain_Amygdala_spredixcan	108	2.176	NA	0.06951	|Z|
KEGG_MAPK_SIGNALING_PATHWAY	MDD	Brain_Anterior_cingulate_cortex_BA24_spredixcan	147	−2.074	NA	0.07366	|Z|
KEGG_RAS_SIGNALING_PATHWAY	MDD	Brain_Anterior_cingulate_cortex_BA24_spredixcan	121	−2.666	NA	0.07409	|Z|
KEGG_SYNAPTIC_VESICLE_CYCLE	MDD	Meta-across-tissues	51	3.410	0.1222	0.1926	|Z|
KEGG_CITRATE_CYCLE_TCA_CYCLE	MDD	Brain_Amygdala_spredixcan	21	2.321	NA	0.2029	|Z|

Appendix 2

**Table 10:** Gene-set relevance ranking across disease combinations

Rank	Gene set	Disease	Composite score	Z	Coverage	Permutation p
1	KEGG_SYNAPTIC_VESICLE_CYCLE	MDD	2.224	3.41	65%	0.1222
2	KEGG_RAS_SIGNALING_PATHWAY	MDD	1.225	−1.74	66%	0.4245
3	KEGG_AUTOPHAGY_ANIMAL	MDD	0.960	−1.26	75%	0.5679
4	KEGG_SYNAPTIC_VESICLE_CYCLE	ALS	0.890	−1.17	68%	0.4924
5	KEGG_NEUROACTIVE_LIGAND_RECEPTOR_INTERACTION	ALS	0.816	1.07	60%	0.5257
6	KEGG_MAPK_SIGNALING_PATHWAY	ALS	0.745	0.93	66%	0.5904
7	KEGG_REGULATION_OF_ACTIN_CYTOSKELETON	MDD	0.735	0.90	70%	0.6815
8	KEGG_FATTY_ACID_DEGRADATION	MDD	0.674	−0.77	79%	0.7244
9	KEGG_AUTOPHAGY_ANIMAL	ALS	0.573	−0.63	71%	0.7052
10	KEGG_ENDOCYTOSIS	ALS	0.498	0.48	76%	0.7852
11	KEGG_ENDOCYTOSIS	MDD	0.443	0.39	78%	0.8604
12	KEGG_CITRATE_CYCLE_TCA_CYCLE	MDD	0.411	0.31	83%	0.8864
13	KEGG_PPAR_SIGNALING_PATHWAY	ALS	0.402	−0.35	71%	0.8367
14	KEGG_FATTY_ACID_DEGRADATION	ALS	0.358	0.22	81%	0.8960
15	KEGG_RAS_SIGNALING_PATHWAY	ALS	0.328	0.24	65%	0.8858
16	KEGG_PPAR_SIGNALING_PATHWAY	MDD	0.316	0.20	71%	0.9251
17	KEGG_NEUROACTIVE_LIGAND_RECEPTOR_INTERACTION	MDD	0.314	−0.24	60%	0.9094
18	KEGG_CITRATE_CYCLE_TCA_CYCLE	ALS	0.302	−0.13	83%	0.9392
19	KEGG_REGULATION_OF_ACTIN_CYTOSKELETON	ALS	0.274	−0.14	68%	0.9376
20	KEGG_MAPK_SIGNALING_PATHWAY	MDD	0.247	−0.10	67%	0.9627

Appendix 3

**Table 11:** Focus-pathway enrichment overview and concordance metrics CCC: Lin’s concordance correlation coefficient; τ: Kendall tau

Focus pathway	ALS Z	MDD Z	Gap	Levene significant	Influential genes	Sign concordance	CCC	τ
KEGG_SYNAPTIC_VESICLE_CYCLE	−1.17	3.41	4.58	No	25	0.431	−0.070	−0.029
KEGG_RAS_SIGNALING_PATHWAY	0.24	−1.74	1.98	Yes	48	0.460	−0.008	0.004
KEGG_NEUROACTIVE_LIGAND_RECEPTOR_INTERACTION	1.07	−0.24	1.31	Yes	190	0.514	−0.062	−0.004
KEGG_REGULATION_OF_ACTIN_CYTOSKELETON	−0.14	0.90	1.04	Yes	123	0.443	0.016	0.000

Appendix 4

**Table 12:** Largest cross-disease signal gaps by pathway

Rank	Gene set	Gap	ALS Z	MDD Z
1	KEGG_SYNAPTIC_VESICLE_CYCLE	4.58	−1.17	3.41
2	KEGG_RAS_SIGNALING_PATHWAY	1.98	0.24	−1.74
3	KEGG_NEUROACTIVE_LIGAND_RECEPTOR_INTERACTION	1.31	1.07	−0.24
4	KEGG_REGULATION_OF_ACTIN_CYTOSKELETON	1.04	−0.14	0.90
5	KEGG_MAPK_SIGNALING_PATHWAY	1.03	0.93	−0.10
6	KEGG_FATTY_ACID_DEGRADATION	0.99	0.22	−0.77
7	KEGG_AUTOPHAGY_ANIMAL	0.63	−0.63	−1.26
8	KEGG_PPAR_SIGNALING_PATHWAY	0.55	−0.35	0.20
9	KEGG_CITRATE_CYCLE_TCA_CYCLE	0.44	−0.13	0.31
10	KEGG_ENDOCYTOSIS	0.09	0.48	0.39

Appendix 5

**Table 13:** Significant pairwise disease statistical tests

Gene set	Contrast	n paired	Mean difference	Cohen d paired	Cohen d unpaired	Levene FDR
KEGG_REGULATION_OF_ACTIN_CYTOSKELETON	MDD vs ALS	151	0.059	0.01	0.05	0.008495
KEGG_ENDOCYTOSIS	MDD vs ALS	186	0.054	0.02	−0.00	0.008495
KEGG_NEUROACTIVE_LIGAND_RECEPTOR_INTERACTION	MDD vs ALS	218	−0.102	−0.05	−0.06	0.009707

Appendix 6

**Table 14:** Family-wise FDR survivors

Family-wise FDR survivor	Test	Raw p	Global FDR
KEGG_REGULATION_OF_ACTIN_CYTOSKELETON	pairwise_stats / Levene p	0.001659	0.03152
KEGG_ENDOCYTOSIS	pairwise_stats / Levene p	0.001699	0.03228

Appendix 7

**Table 15:** Null or absent result classes

Analysis section	Significant findings	Interpretation
Differential tests: Kruskal-Wallis and pairwise	0	No significant differential results at the specified thresholds.
Profile proximity: Pearson / Spearman	0	No significant proximity pairs at the specified thresholds.
Cross-disease concordance / discordance	0	No shared or opposed directional programs passed concordance thresholds.
Concordance metrics: sign / CCC / Kendall τ	0	No significant concordance results at the specified thresholds.

Appendix 8

**Table 16:** Most influential genes across all gene sets, ranked by flag frequency and mean absolute Stouffer influence Note. The complete run flagged 1024 influential gene-disease entries; the downstream summary collapsed these to 756 unique genes.

Rank	Gene	Flagged entries	Gene sets	Mean |ΔStouffer|	Sign-flip noted
1	EGF	5	3	0.370	Yes
2	FGF8	5	3	0.222	Yes
3	FGFR4	5	3	0.192	No
4	AKT3	5	3	0.189	Yes
5	ACSL5	4	2	0.637	Yes
6	ACOX1	4	2	0.360	No
7	ACSL1	4	2	0.358	Yes
8	PCK2	4	2	0.311	Yes
9	UBC	4	2	0.297	Yes
10	KNG1	4	2	0.293	Yes
11	CPT1B	4	2	0.256	Yes
12	CPT2	4	2	0.241	Yes
13	PCK1	4	2	0.212	Yes
14	ARPC1A	4	2	0.191	No
15	ACSBG2	4	2	0.166	Yes
16	ACADM	4	2	0.138	No
17	MAPK1	4	4	0.134	No
18	RAF1	4	4	0.122	No
19	RRAS2	3	3	0.337	Yes
20	PLA2G4B	3	2	0.319	No
21	CPT1C	3	2	0.274	Yes
22	RASA2	3	2	0.272	Yes
23	GNA12	3	2	0.247	No
24	NTRK1	3	2	0.239	No
25	RAC3	3	2	0.230	No

Appendix 9

**Table 17:** Genes influential across at least two focus pathways

Rank	Gene	No. pathways	Pathways	Mean |Δ|	Sign flip
1	EGF	2	RAS signaling; regulation of actin cytoskeleton	0.399	Yes
2	KNG1	2	Neuroactive ligand-receptor interaction; regulation of actin cytoskeleton	0.293	Yes
3	FGF8	2	RAS signaling; regulation of actin cytoskeleton	0.229	No
4	FGF17	2	RAS signaling; regulation of actin cytoskeleton	0.155	No
5	RAC1	2	RAS signaling; regulation of actin cytoskeleton	0.151	No
6	FGFR1	2	RAS signaling; regulation of actin cytoskeleton	0.143	No
7	MAPK1	2	RAS signaling; regulation of actin cytoskeleton	0.142	No
8	BDKRB1	2	Neuroactive ligand-receptor interaction; regulation of actin cytoskeleton	0.142	Yes
9	PIK3R3	2	RAS signaling; regulation of actin cytoskeleton	0.141	No
10	PAK2	2	RAS signaling; regulation of actin cytoskeleton	0.127	No
11	RAF1	2	RAS signaling; regulation of actin cytoskeleton	0.125	No
12	PAK1	2	RAS signaling; regulation of actin cytoskeleton	0.124	No
13	BDKRB2	2	Neuroactive ligand-receptor interaction; regulation of actin cytoskeleton	0.096	No
14	CHRM3	2	Neuroactive ligand-receptor interaction; regulation of actin cytoskeleton	0.062	No

Appendix 10

**Table 18:** Stage 3 per-pathway influential genes reported for focus pathways Note. This table contains the Stage 3 focus-pathway influential gene listings reported in the supplied summary. Duplicate entries for VAMP2 and ATP6V0B in the original synaptic vesicle listing were consolidated to preserve one row per gene-level comparison.

Pathway	Gene	Z	ΔStouffer	% change	MDD Z	ALS Z	Divergence	Sign flip
Synaptic vesicle cycle	ATP6V0D1	7.64	1.058	125.0	3.27	7.64	4.38	No
Synaptic vesicle cycle	VAMP2	−5.32	−0.723	85.4	7.97	−5.32	13.29	No
Synaptic vesicle cycle	ATP6V1D	−4.52	−0.613	72.5	−1.25	−4.52	3.27	No
Synaptic vesicle cycle	ATP6V0A1	−3.53	−0.476	56.3	2.48	−3.53	6.01	No
Synaptic vesicle cycle	ATP6V1E2	−3.17	−0.427	50.5	2.02	−3.17	5.18	No
Synaptic vesicle cycle	DNM3	−3.05	−0.411	48.5	0.59	−3.05	3.63	No
Synaptic vesicle cycle	NAPA	−2.80	−0.377	44.6	−0.97	−2.80	1.83	No
Synaptic vesicle cycle	CLTA	2.41	0.338	40.0	−0.11	2.41	2.51	No
Synaptic vesicle cycle	STX1A	−2.05	−0.273	32.3	−2.63	−2.05	0.58	No
Synaptic vesicle cycle	CPLX2	1.81	0.257	30.4	−0.69	1.81	2.50	No
Synaptic vesicle cycle	SLC6A7	1.72	0.244	28.9	0.22	1.72	1.50	No
Synaptic vesicle cycle	SLC1A2	1.68	0.238	28.2	−0.39	1.68	2.06	No
Synaptic vesicle cycle	CACNA1B	1.61	0.230	27.1	NA	1.61	NA	No
Synaptic vesicle cycle	STX1B	−1.73	−0.229	27.1	−1.84	−1.73	0.11	No
Synaptic vesicle cycle	STX3	1.57	0.224	26.4	3.79	1.57	2.21	No
Synaptic vesicle cycle	ATP6V1C2	−1.63	−0.216	25.5	−1.06	−1.63	0.58	No
Synaptic vesicle cycle	ATP6V0A4	1.41	0.202	23.9	−0.97	1.41	2.38	No
Synaptic vesicle cycle	CLTCL1	1.39	0.199	23.5	−3.26	1.39	4.65	No
Synaptic vesicle cycle	CLTC	1.31	0.188	22.3	−0.94	1.31	2.26	No
Synaptic vesicle cycle	ATP6V1E1	−1.42	−0.188	22.2	1.17	−1.42	2.59	No
Synaptic vesicle cycle	DNM1	−1.39	−0.183	21.6	3.49	−1.39	4.88	No
Synaptic vesicle cycle	ATP6V0B	1.24	0.179	21.1	5.39	1.24	4.14	No
Synaptic vesicle cycle	ATP6V1G2	1.20	0.173	20.4	1.91	1.20	0.72	No
RAS signaling	TBK1	11.23	0.903	145.3	−2.89	11.23	14.12	Yes
RAS signaling	LAT	−6.51	−0.526	84.7	−0.51	−6.51	6.00	No
RAS signaling	EGF	−5.73	−0.463	74.6	−3.46	−5.73	2.27	No
RAS signaling	PLA2G12A	5.38	0.431	69.4	0.49	5.38	4.89	No
RAS signaling	ZAP70	−5.14	−0.416	67.0	1.46	−5.14	6.60	No
RAS signaling	RAB5C	5.13	0.412	66.3	−0.68	5.13	5.81	No
RAS signaling	GNG10	4.86	0.389	62.7	−0.42	4.86	5.27	No
RAS signaling	NTRK1	−4.44	−0.360	57.9	0.54	−4.44	4.98	No
RAS signaling	CHUK	4.30	0.345	55.5	0.10	4.30	4.21	No
RAS signaling	RASA2	−3.86	−0.313	50.4	−3.20	−3.86	0.66	No
RAS signaling	BCL2L1	−3.75	−0.305	49.0	0.27	−3.75	4.02	No
RAS signaling	PLA2G3	−3.60	−0.292	47.0	0.88	−3.60	4.48	No
RAS signaling	EFNA1	3.58	0.287	46.2	0.72	3.58	2.86	No
RAS signaling	RAB5B	3.50	0.280	45.0	1.30	3.50	2.19	No
RAS signaling	ANGPT1	3.37	0.270	43.4	−0.54	3.37	3.91	No
RAS signaling	RASSF5	2.93	0.234	37.6	−2.36	2.93	5.28	No
RAS signaling	RASA1	−2.84	−0.231	37.2	−2.95	−2.84	0.10	No
RAS signaling	GNG5	−2.81	−0.229	36.8	−3.49	−2.81	0.68	No
RAS signaling	KITLG	2.68	0.214	34.4	3.87	2.68	1.20	No
RAS signaling	SHOC2	−2.60	−0.212	34.1	−3.47	−2.60	0.87	No
RAS signaling	FGF8	2.65	0.211	34.0	−3.31	2.65	5.96	No
RAS signaling	ANGPT2	−2.37	−0.193	31.1	0.34	−2.37	2.71	No
RAS signaling	GNB2	−7.47	−0.590	30.3	−7.47	1.46	8.93	No
RAS signaling	NF1	2.31	0.184	29.6	−0.28	2.31	2.59	No
RAS signaling	RASAL1	−2.17	−0.177	28.5	−2.23	−2.17	0.05	No
Neuroactive ligand-receptor interaction	HTR1B	7.36	0.493	434.9	7.36	−0.73	8.09	No
Neuroactive ligand-receptor interaction	HTR6	6.01	0.403	355.4	6.01	2.58	3.43	No
Neuroactive ligand-receptor interaction	P2RY6	−5.62	−0.376	331.7	−5.62	−0.52	5.10	Yes
Neuroactive ligand-receptor interaction	GAL	4.97	0.333	293.9	4.97	−2.06	7.03	No
Neuroactive ligand-receptor interaction	CHRNB1	−4.85	−0.324	286.1	−4.85	−0.25	4.59	Yes
Neuroactive ligand-receptor interaction	MCHR1	−4.20	−0.281	247.9	−4.20	2.44	6.64	Yes
Neuroactive ligand-receptor interaction	GRIK2	−4.02	−0.269	237.3	−4.02	0.23	4.25	Yes
Neuroactive ligand-receptor interaction	NMUR2	4.00	0.268	236.4	4.00	−5.50	9.50	No
Neuroactive ligand-receptor interaction	NPFF	−4.00	−0.268	236.1	−4.00	−0.98	3.02	Yes
Neuroactive ligand-receptor interaction	TSHR	3.97	0.266	234.7	3.97	−1.01	4.98	No
Neuroactive ligand-receptor interaction	AVP	3.80	0.255	224.5	3.80	2.61	1.18	No
Neuroactive ligand-receptor interaction	KNG1	−3.75	−0.251	221.6	−3.75	4.25	8.00	Yes
Neuroactive ligand-receptor interaction	FSHB	−3.60	−0.241	212.7	−3.60	−1.13	2.47	Yes
Neuroactive ligand-receptor interaction	GRIN1	3.59	0.241	212.2	3.59	−1.46	5.05	No
Neuroactive ligand-receptor interaction	C5AR1	3.53	0.236	208.6	3.53	0.15	3.38	No
Neuroactive ligand-receptor interaction	UCN2	3.38	0.227	200.1	3.38	−1.22	4.61	No
Neuroactive ligand-receptor interaction	P2RY11	3.36	0.225	198.9	3.36	0.81	2.56	No
Neuroactive ligand-receptor interaction	CHRNA4	3.32	0.223	196.4	3.32	NA	NA	No
Neuroactive ligand-receptor interaction	PTGFR	−3.26	−0.218	192.4	−3.26	0.46	3.72	Yes
Neuroactive ligand-receptor interaction	CHRNE	−3.16	−0.211	186.4	−3.16	−2.10	1.06	Yes
Neuroactive ligand-receptor interaction	GRM6	−3.08	−0.206	182.0	−3.08	−1.18	1.90	Yes
Neuroactive ligand-receptor interaction	PRSS3	−3.01	−0.202	177.8	−3.01	3.34	6.36	Yes
Neuroactive ligand-receptor interaction	EDN3	−3.01	−0.201	177.4	−3.01	0.25	3.25	Yes
Neuroactive ligand-receptor interaction	GALR1	2.97	0.199	175.9	2.97	1.50	1.47	No
Neuroactive ligand-receptor interaction	MCHR2	−2.89	−0.193	170.3	−2.89	1.23	4.12	Yes
Regulation of actin cytoskeleton	EGF	−5.73	−0.457	151.2	−3.46	−5.73	2.27	Yes
Regulation of actin cytoskeleton	KNG1	4.25	0.341	112.8	−3.75	4.25	8.00	No
Regulation of actin cytoskeleton	ITGA3	4.04	0.325	107.3	−1.66	4.04	5.70	No
Regulation of actin cytoskeleton	PPP1R12B	3.13	0.252	83.3	−0.08	3.13	3.21	No
Regulation of actin cytoskeleton	ITGA6	2.98	0.239	79.1	0.56	2.98	2.41	No
Regulation of actin cytoskeleton	LIMK2	2.90	0.233	77.1	−1.61	2.90	4.51	No
Regulation of actin cytoskeleton	WASL	2.87	0.231	76.3	1.85	2.87	1.02	No
Regulation of actin cytoskeleton	ARPC5	2.71	0.218	71.9	−2.03	2.71	4.73	No
Regulation of actin cytoskeleton	FGF8	2.65	0.213	70.4	−3.31	2.65	5.96	No
Regulation of actin cytoskeleton	ITGA1	−2.67	−0.213	70.4	0.15	−2.67	2.82	No
Regulation of actin cytoskeleton	FN1	2.60	0.209	69.2	−4.65	2.60	7.26	No
Regulation of actin cytoskeleton	ARPC2	−2.44	−0.194	64.2	−1.64	−2.44	0.80	No
Regulation of actin cytoskeleton	ITGB3	−2.38	−0.190	62.7	−1.52	−2.38	0.86	No
Regulation of actin cytoskeleton	WASF2	2.17	0.175	57.8	2.37	2.17	0.20	No
Regulation of actin cytoskeleton	PPP1CB	8.70	0.679	57.7	8.70	0.92	7.77	No
Regulation of actin cytoskeleton	ITGAD	2.16	0.174	57.5	0.38	2.16	1.78	No
Regulation of actin cytoskeleton	MYL12B	−2.16	−0.172	56.9	1.44	−2.16	3.60	No
Regulation of actin cytoskeleton	MYLK2	2.02	0.162	53.7	−0.38	2.02	2.39	No
Regulation of actin cytoskeleton	ARPC1A	2.01	0.162	53.5	3.08	2.01	1.07	No
Regulation of actin cytoskeleton	FGF17	1.94	0.156	51.6	−1.75	1.94	3.68	No
Regulation of actin cytoskeleton	ROCK1	1.93	0.156	51.5	−1.42	1.93	3.35	No
Regulation of actin cytoskeleton	MYL9	−1.95	−0.155	51.3	1.60	−1.95	3.55	No
Regulation of actin cytoskeleton	ACTR3C	−1.94	−0.154	51.1	−1.29	−1.94	0.66	No
Regulation of actin cytoskeleton	SCIN	−1.92	−0.152	50.4	1.07	−1.92	2.98	No
Regulation of actin cytoskeleton	RAC1	−1.88	−0.149	49.4	0.31	−1.88	2.19	No

Appendix 11

Availability of Data and Materials

MDD GWAS summary statistics are publicly available through the Psychiatric Genomics Consortium repository. ALS summary statistics are available through Project MinE. The NMN aging dataset, GSE85718, is accessible through the Gene Expression Omnibus. GTEx-derived S-PrediXcan tissue models and prediction resources are publicly distributed. The derived files generated in the current analysis included multi-gene-set enrichment summaries, pairwise disease statistics, gene-influence tables, per-gene Z-score tables, family-wise FDR tables, run manifests, and detailed summary files. At the time of writing, the derived outputs and input identifiers are available from the corresponding author upon reasonable request.

The exact PredictDB model release, orthology release, and package-version manifest were not retained in the original run. Public deposition of the source code, input gene-set collection, run manifest, and derived outputs in a version-controlled repository with an archival DOI is planned before publication.

Appendix 12

Code Availability

Analysis code implementing cross-tissue Stouffer meta-Z aggregation, competitive permutation testing, Wilcoxon signed-rank tests, Levene variance testing, leave-one-out influence analysis, concordance metrics, multi-gene-set processing, and downstream summary generation is available from the corresponding author upon reasonable request. The code uses Python with pandas, NumPy, SciPy, matplotlib, seaborn, and NetworkX.
